# PET/CT and brown fat in the evaluation of treatment response in Hodgkin lymphoma

**DOI:** 10.1590/0100-3984.2015.0029

**Published:** 2015

**Authors:** Laís Bastos Pessanha, André Ribeiro Nogueira de Oliveira, Luiz Felipe Alves Guerra, Diego Lima Nava Martins, Ronaldo Garcia Rondina, Melissa Bozzi Nonato Mello

**Affiliations:** 1 Universidade Federal do Espírito Santo (UFES), Vitória, ES, Brazil.

*Dear Editor,*

**Figure 1:**
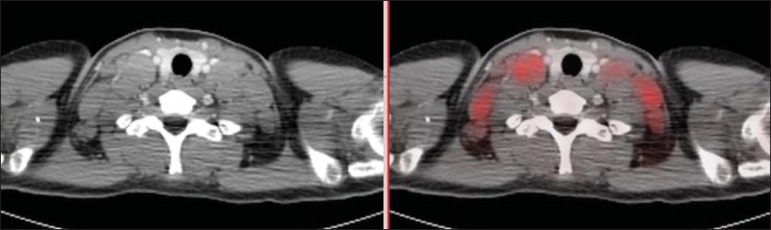
Pre-chemotherapy PET/CT image showing hypermetabolic lymph node enlargement in the cervical chains.

A female, 15-year-old patient presented with insidious onset of weight loss and low fever. Hodgkin's lymphoma was diagnosed after biopsy of a palpable enlarged lymph node. ^18^F-FDG PET/CT was performed during the initial staging, demonstrating hypermetabolic mediastinal, axillary and cervical lymph node enlargement (Figure 1). The findings were interpreted as lymphoma in activity in the mentioned sites. At basal PET/CT study, one could not observe metabolic activity in brown fat. Chemotherapy was initiated with adriblastine, bleomycine, vinblastine and dacarbazine at days D1 and D15 for every 28-day cycles.

**Figure 2:**
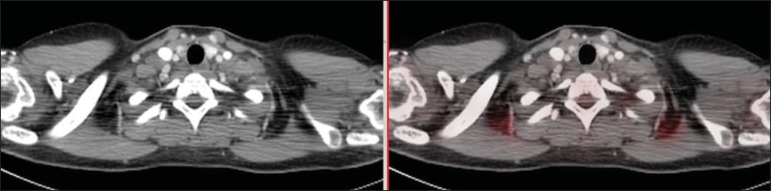
Follow-up PET/CT after three-month chemotherapy demonstrating onset of activity in adipose tissue with typical distribution of brown fat.

Six chemotherapy cycles were uneventfully performed. A new FDG PET/CT performed after about three months to evaluate the therapeutic response demonstrated complete regression of all the lesions interpreted as lymphoma in activity at the first study. Also, the onset of activity was observed in fat tissue with typical brown fat distribution (at the neck base and shoulders - Figure 2).

Brown fat is an adipose tissue specialized in the generation of heat by means of glucose metabolization (differently from the white fat whose function is just storing energy under the form of lipids)^(1,2)^.

As compared with white fat, brown fat has abundant vascularization and innervation by the sympathetic nervous system. Many times, the metabolic activity in the brown fat may obscure intermingled hypermetabolic lesions (metastatic lymph nodes, for example)^(1,2)^.

The onset of activity in brown fat with disappearance of lesions suspicious for active lymphoma after chemotherapy completion in children and adolescents is described in the literature and is related to a complete therapeutic response in the lymphoma^(3,4)^. Such an inverse relationship between the absence of tumor and presence of brown adipose tissue has been observed in both female and male patients regardless their body mass index and temperature^(3,4)^. The possible mechanisms for brown fat suppression by the lymphoma still remain unknown. However, patients with malignant lymphomas present with high levels of tumor necrosis factor alpha, an important cytokine capable of inducing a great number of biological effects in multiple systems, including apoptotic degeneration of brown adipocytes^(1-4)^.

PET/CT using FDG has been widely adopted as the main imaging modality in the evaluation of lymphomas^(5,6)^. The identification of brown adipose tissue in humans by PET/CT has revived the interest in the function and relevance of those cells, since there was a concept that they were seen only in neonates, and currently they are identified by PET/CT also in children and young adultsv^7,8)^. The knowledge that the brown adipose tissue is a predictor of disease state contributes to a correct analysis of images from children and adolescents with lymphoma, being useful in the follow-up and clinical therapeutics of those patients.
